# Copy Number Variants in Four Italian Turkey Breeds

**DOI:** 10.3390/ani11020391

**Published:** 2021-02-03

**Authors:** Maria Giuseppina Strillacci, Stefano Paolo Marelli, Raffaella Milanesi, Luisa Zaniboni, Chiara Punturiero, Silvia Cerolini

**Affiliations:** Department of Veterinary Medicine, University of Milan, Via dell’Università 6, 26900 Lodi, Italy; stefano.marelli@unimi.it (S.P.M.); raffaella.milanesi@unimi.it (R.M.); luisa.zaniboni@unimi.it (L.Z.); chiara.punturiero1@studenti.unimi.it (C.P.); silvia.cerolini@unimi.it (S.C.)

**Keywords:** Copy Number Variant, Turkey breeds, CNV, autochthonous populations

## Abstract

**Simple Summary:**

Hybrid Turkey selection is focusing on meat production traits characterized by high genetic heritability; the strong directional selection is well known to produce a constant loss in genetic diversity. Genetic characterization is one of the essential activities in the management of populations at risk of extinction. In addition, the genetic structure at the population level and the relationships between individuals are nowadays analysable at the genomic level. In this paper, the genome of 4 different Italian turkey breeds included in the Autochthonous Italian Poultry Breeds Register are analysed in order to obtain a genome-wide Copy Number Variant scan to ameliorate the existing knowledge of the genomic structure of Italian local turkey breeds. Differences have been described at genomic level for physiological, reproductive, and behavioral traits. The analyzed breeds are clearly distinguishable at the genomic level, and their relationships are clearly linked to their geographical origin and to the history of the rural structure of their developing regions. Genome information based on Copy Number Variant (CNV) detection has generated important information in this study concerning the uniqueness of the Italian local turkey breeds.

**Abstract:**

Heritage breeds can be considered a genetic reservoir of genetic variability to be conserved and valorized considering their historical, cultural, and adaptive characteristics and possibly for their high potential in commercial hybrid genetic improvement by gene introgression. The aim of the present research is to investigate via Copy Number Variant (CNVs) the genomic makeup of 4 Italian autochthonous turkey breeds (Bronzato Comune—BrCI, 24; Ermellinato di Rovigo—ErRo, 24; Parma e Piacenza—PrPc, 25; Romagnolo—RoMa, 29). CNVs detection was performed using two different software and an interbreed CNVs comparison was carried out. A total of 1077 CNVs were identified in 102 turkeys, summarized into 519 CNV regions (CNVRs), which resulted after merging in 101 and 18 breed and shared regions. Biodiversity was analyzed using the effective information supplied by CNVs analysis, and BrCI and ErRo were characterized by a low mapped CNV number. Differences were described at a genomic level related to physiological, reproductive, and behavioral traits. The comparison with other three Italian turkey breeds (Brianzolo, Colle Euganei, and Nero Italiano) using a CNV data set available in the literature showed high clustering properties at the genomic level, and their relationships are strictly linked to the geographical origin and to the history of the rural structure of their native regions.

## 1. Introduction

The turkey industry is oriented to high-quality low-cost meat production based on highly performing commercial hybrids mainly selected for a balanced breeding goal, including production traits such as reproductive performance, growth, feed conversion efficiency, yield, and functional and health traits. Several authors have studied the effect of heavy selection on functional characteristics of birds such as immune system efficacy, oxidative stress resistance, and cardio-circulatory system functionality [1,2,3,4,5]. The Mexican Turkey (*Meleagris gallopavo gallopavo*), domesticated almost 3000 years ago, is the wild ancestor of domestic turkey heritage breeds and commercial hybrids [6]. Livestock domestication and captive evolution resulted in a variety of phenotypes performing high adaptability and coping ability to specific environments and rearing conditions. The increase in the production through selection for economically important traits characterized by high genetic heritability has been correlated to a constant loss in genetic diversity [7]. 

Genetic diversity is the basic requirement for both genetic improvement and response to selection objectives [8]. In turkeys, heritage breeds can be considered a genetic reservoir of genetic variability to be conserved and valorized considering their historical, cultural, and adaptive characteristics and their high potential in commercial hybrid genetic improvement by gene introgression [5,9]. 

Italy is one of the richest countries in biodiversity, and currently, eight breeds are actively bred under the control of the Italian Ministry of Agriculture (Mipaaf). Autochthonous turkey breeds are reared in Italy in rural and family farms, and the size of the populations is presently very limited. The inclusion of these breeds in conservation programs is essential for their safeguard and for the conservation of their characteristics: morphology, reactivity, high adaptation to the environment, and disease resistance [10]. 

In recent years, many efforts to include local avian breeds within conservation programs have been realized with the support of regional and national institutions with an important poultry tradition. Among these programs, the TuBAvI project— “Conservation of biodiversity in Italian poultry breeds” was funded by EAFRD (European Agricultural Fund for Rural Development) [11]. The aim of TuBAvI project was the “safeguard, the conservation and the improvement of the Italian poultry genetic resources, represented by many autochthonous breeds historically present in the Country”. 

In addition, in this context, in 2014 (Mipaaf - DM 19536, 1 October 2014), the Registro Anagrafico delle Razze Avicole Autoctone (RAA; Autochthonous Italian Poultry Breeds Registry) was established. It follows the effort of the national legislation in adopting international guidelines aimed to preserve Animal Genetic Resources in avian species. Eight autochthonous Italian turkey breeds (Brianzolo, Bronzato Comune, Castano Precoce, Colli Euganei, Ermellinato di Rovigo, Nero d’Italia, Parma e Piacenza, Romagnolo) are listed in the RAA.

Genetic characterization is one of the essential activities in the management of populations at risk, and thanks to the recent availability of high-throughput genotyping techniques, also for the poorly studied species, the genetic structure at the population level and the relationships among breeds and between individuals are nowadays analysable at genomic level [7].

The genetic variability in turkey populations has been recently studied mainly using Single Nucleotide Polymorphisms (SNPs) as markers [12,13,14,15] and by the analysis of mtDNA D-loop sequences [16]. Copy number variants (CNVs) in turkeys species are still poorly studied [17,18,19]. CNVs are DNA segments longer more than 50 bp (deleted or duplicated) present at a variable copy number in comparison with a reference genome and affecting gene expression [20]. As shown in recent literature, CNVs can contribute to understanding the differences both at individual and populations level, in disease susceptibility [21,22], phenotypic, and genome variability also as a response to selection pressure [23,24,25,26].

In this paper, the genome of four different Italian breeds, part of the RAA, have been analyzed in order to obtain a genome-wide copy number scan and to ameliorate the existing knowledge of genomic structure of Italian local turkey breeds.

## 2. Materials and Methods

### 2.1. Sampling and Genotyping

The genotypes of 12 birds of Parma e Piacenza (PrPc) breed from a previous study [18] are complemented in this study with 13 birds of the same breed and 78 individuals of 3 additional Italian turkey breeds (Bronzato Comune: BrCI, Ermellinato di Rovigo: ErRo, and Romagnolo: RoMa) were collected for this study within the activity of the TuBAvI project (Table 1).

DNA was extracted from collected blood turkey samples using ZR Genomic DNA TM Tissue MiniPrep (Zymo, Irvine, CA, U.S.A.). DNA samples were quantified using NanoQuant Infinite^®^m200 (Tecan, Männedorf, Switzerland), diluted to 40 ng/μL and genotyped with Axiom^®^ Turkey Genotyping Array (Affymetrix), containing 634,067 SNPs mapped on the Turkey_5.01 (GCA_000146605.4) genome assembly.

Using the standard protocol in the Affymetrix Power Tools package [27], a quality control of raw intensity files was performed. Default quality control parameters were applied to filter for low-quality samples, i.e., genotyping call rate <98% and Dish Quality Control <0.82.

### 2.2. CNVs and CNVRs Detection

The CNVs detection was performed on autosomes (from 1 to 30) using the same approaches employed in [18]. The Log R Ratio (LRR) and the B allele frequency (BAF) values were obtained using the Axiom^®^ CNV Summary Tool software. 

Two different calling algorithms were considered in order to reduce the false-positive calls: (i) the univariate analysis in the Copy Number Analysis Module of SVS 8.8.4 software of Golden Helix [28] and (ii) the Hidden Markov Model of PennCNV software v1.04 [29], using three different “hmm” files: agre.hmm and affygw6.hmm (both specific for Affymetrix SNP array) and hh550.hmm (developed for Illumina SNP arrays and considered in this study because it is based on an SNPs chip density closest to the one used here). Outlier samples for LRR were identified before the SVS CNV detection through (i) the overall distribution of Derivative Log Ratio Spread (DLRS) values and (ii) the GC content screening, which is correlated to a long-range waviness of LRR values by the wave detection factor algorithm as in [30]. 

The CNV calling has been obtained by SVS using the univariate analysis based on LRR values, with the following options: univariate outlier removal, a limit of not more than 100 segments per 10,000 markers with a minimum of three markers per segment, and 2000 permutations per pair with a *p*-value cut off of 0.005. 

The PennCNV calling was based on LRR and BAF values using the default parameters: standard deviation of LRR <0.30, BAF drift as 0.01 and waviness factor at 0.05, with a minimum number of three consecutive SNPs required to define a CNV. 

A consensus analysis was performed at the individual level with the -intersectBed command of the BEDTools software [31], using the fully overlapping CNVs (in terms of genome position and CNV state—loss and gain), among those identified in at least two detection approaches (CNVs).

CNV regions (CNVRs) were defined at the breed level (i.e., within each breed) and at the overall level (i.e., between breeds). CNVRs were identified by merging CNVs identified in at least two birds using the -mergeBed command of BEDTools [31]. CNVRs were classified in concordance with the regions state in gain (all CNVs gain), loss (all CNV loss), and complex (CNVs both gain and loss). CNVs identified in only one individual were considered singleton_CNVRs.

Comparing CNVRs across breeds, shared_CNVRs have been defined through the -intersectBed command of BEDTools as those mapped in at least 2 breeds. The CNVRs found in only one breed were defined as breed_CNVRs.

Genes were annotated within the CNVRs using the NCBI Turkey_5.0 gene dataset (annotation Release 103), and the BEDTools “-intersectBed” command [31] was used to catalogue these genes to the corresponding regions (excluding singleton_CNVRs). Gene Ontology terms (GO) and Kyoto Encyclopedia of Genes and Genomes (KEGG) pathway analyses were performed using the DAVID Bioinformatic Database [32]. Only LOC genes catalogued in NCBI Database as protein genes were considered, and the uncharacterized “LOC” protein genes were then excluded. 

In addition, the National Animal Genome Research Program database was utilized using the “Search by associated gene” option in order to identify QTL-associated traits for genes annotated within CNVRs. Considering that there is no specific QTL database for the Turkey species, we accessed the one available for chicken (Chicken QTLdb, *Gallus gallus* 5.0—GG5.0) [33]. 

### 2.3. Interbreeds Comparison Analysis

In order to disclose genomic structure diversity among these turkey breeds, a matrix was built attributing “0” to absence of CNV in a CNVR (normal state), “1” to deletions and “2” to duplications. The matrix has been used as input of PAST 3.22 [34] software to perform a Principal Component Analysis (PCA).

### 2.4. Comparison with Literatures

CNVs and the CNVRs detected in this study have been compared with results available in the literature on turkey populations [18]. The published CNV mapping results for the Brianzolo (BR), Colli Euganei (CoEu), Nero Italiano (NI), available from the additional files [18], was used to integrate the CNV and CNVR mapped in this study and jointly used in a PCA, performed using the same approach described in the previous subsection.

## 3. Results

### 3.1. Data Editing, CNVs, and CNVRs Detection

Only one sample (RoMa breed) was excluded during quality assurance for its high DLRS value. The total number of CNVs resulted after the consensus analysis was 1077 and, as reported in Table 2, varied in terms of number and size among the individuals of each population. The lowest number of CNVs was identified for the BrCI and ErRo breeds, as well as the lower mean length. For these two breeds, the resulting Loss/Gain ratios (calculated as the total number of Loss CNV on Total CNV number) were inverted and higher respect to those identified for the other breeds. Each breed_CNVs dataset is reported in Table S1.

**Figure 1:**
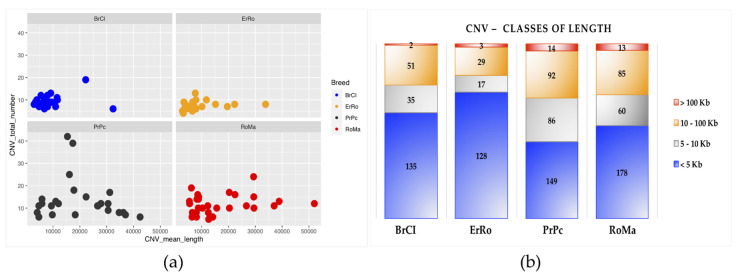
Graphical representation of CNVs statistics: (**a**) relationship between samples CNVs number and mean CNV length (bp) per birds; (**b**) number of CNVs for each class of length per breed.

The graphical overview of the relationship existing between the CNV count and the mean total length of CNVs are visualized in Figure 1. In general (Figure 1a), the majority of samples (colored dots—each dot represents an individual) had a low number of CNVs with a high variability in averaged length, except for sample of PrPc showing a large variability in number as well as in CNV length. 

When CNVs are classified according to classes of length (four classes—Figure 1b), all breeds showed different characteristics, even if, as expected, the largest CNVs class is the short one (< 5 Kb) for all breeds (mainly in ErRo).

The CNVs—across samples—were summarized into 519 copy number variable regions (CNVRs), when overlapping across individuals. The descriptive statistics of breed_CNVR are reported in Table 3. Table S2 includes the complete list of the CNVRs detected per breed.

The BrCI and ErRo breeds showed a low number of total CNVRs and of the singleton_CNVRs in comparison to other ones (Table 3). In addition, the number of gain and loss regions are similar and no complex CNVRs were identified for these two breeds. The proportion of the genome covered by CNVRs ranged from 0.07% (ErRo) to 0.32% (PrPc).

CNVRs were found on all autosomes, except on chr 18. Excluding singleton_CNVRs (n. 371), the remaining regions (n. 147) are mapped on 21 chrs (none on autosomes 15, 17, 18, 20, 22–25, 27): (i) the largest proportion of regions were identified on the first five chrs (n. 87–73%), and (ii) on the 19 remaining autosomes, the number of mapped CNVR is low (from 1 to 5). The CNVRs length is variable, ranging from a minimum of 0.82 Mb in chr13 to a maximum of about 45 Mb in chr3.

When merged, the 147 CNVRs (Table S2) resulted in 119 regions consituted of 18 shared_CNVRs and 101 breed_CNVRs (n. 24—BrCI; n. 13—ErRo; n. 28—PrPc; n. 36—RoMa). Table S3 reports the breed_CNVRs with the annotated genes and the associated traits, if available online.

The Table 4 reports details of the CNVRs (n. 17) detected in at least 10 samples (considering all samples of the 4 breeds) and the annotated genes (n. 21): 11 and 6 were shared_CNVRs and breed_CNVRs, respectively. Two flanking regions, all mapped on chr4, are in common to all breeds (CNVR_092, CNVR_093) and are shared by 44 and 41 individuals respectively, including all ErRo birds and a variable number of samples of the other breeds. On the same autosome, the CNVR_096 has also been mapped in all breeds (n. 28 birds). As shown in Table 4, the most represented breed_CNVRs that have been identified are: CNVR_099 – ErRo (n. 16; corresponding to 66% of ErRo birds) and CNVR_061 – RoMa (n. 19; corresponding to 65% of RoMa birds). 

Among the 119 CNVRs, only 38 regions harboured protein-coding genes (total number of annotated genes = 54). Table S4 reports the results of enrichment gene analysis (all 54 genes were included in a unique list given the low number) performed with DAVID Database using Meleagris gallopavo as background species. Among the 50 recognized official gene IDs, none were significantly enriched (nominal *p*-value < 0.05, 10 Terms resulted—Table S4), maybe because of the scarce number of uploaded genes in the annotation analysis.

### 3.2. Interbreeds Comparison Analysis

**Figure 2:**
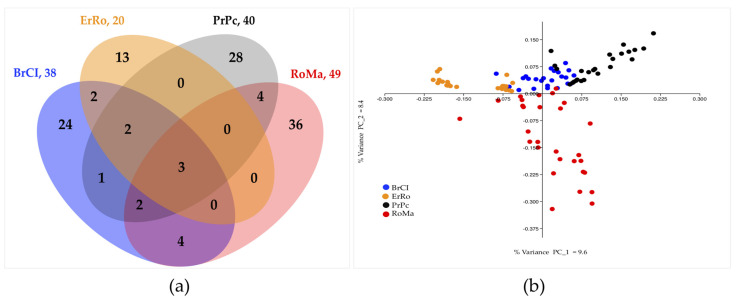
(**a**) Venn diagram of shared and breed CNVRs (n. 147 in total) of each turkey breed here analyzed; (**b**) Principal Component Analysis result; PC_1 vs. PC_2 percentage variance values are plotted.

The Venn diagram reported in Figure 2a shows the number of shared and breed_CNVRs identified for the four breeds (n. 147 in total). PCA analysis shows a spatial distribution of samples according to breed (Figure 2b). In addition, in the overlapping graphic area of the figure samples are distributed according to the number of regions they shared. 

### 3.3. Comparison with Literatures

**Figure 3:**
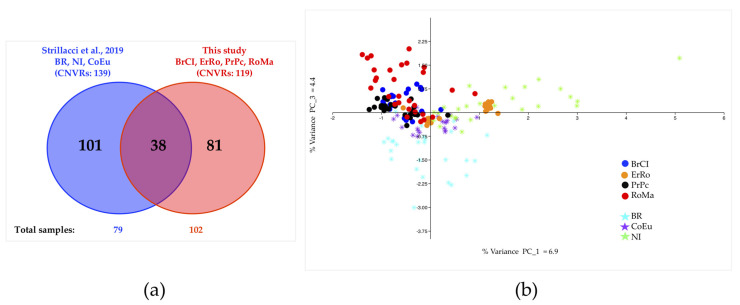
Comparison of seven Italian turkey breeds. (**a**) Venn Diagram of shared and breed CNVRs of the Italian Turkey breeds here analyzed (BrCI, ErRo, PrPc, and RoMa) and identified by Strillacci et al., 2019 (Brianzolo—BR, Colle Euganei—CoEu, and Nero Italiano—NI); (**b**) PCA result for the same seven breeds; PC_1 vs. PC_3 percentage variance values are plotted.

Among the 119 CNVRs (here identified in at least two samples), 38 (31.9%) regions were already mapped in other three Italian turkey breeds (Figure 3a).

The CNVR integrated dataset (n. 220 CNVRs from this study and [18]) used in this comparison (Table S2) comprised 46 shared_CNVRs and 174 breed_CNVRs (Table S5).

Figure 3b shows the distribution of samples that appear to reflect the phenotypical selection target (feather color) and geographical origins.

## 4. Discussion

The Italian populations are the result of a phenotypic selection operated by individual farmers in their small group of individuals to obtain birds that best perform in a semi-extensive farming system. These systems are characterized for a backyard with recovery availability and feeding supplement that was a common practice in the middle ages poultry system of Italy. The present research is part of the TuBAvI poultry genetic resources conservation project envisaging the sampling of birds belonging to breeds, part of the Italian Ministry of Agriculture Register for Italian Poultry Breeds. The unique genomic biodiversity of traditional Italian poultry breeds is a unique resource for the investigation of genetic variation in turkeys with the aim to explore the intimate links occurring among animal breeding, adaptability with specific environments, and animal product characteristics [35,36]. We investigated here the genomic structure of the Italian turkeys through the analysis of the CNVs in order to provide information that could be used to assess biodiversity among breeds and provide basic knowledge for in situ conservation breeding programs of these populations.

A total of 1077 CNVs corresponding to 519 CNVRs were found in this study. Generally, for the BrCI and ErRo, the number of CNVs per bird was different from other breeds. Particularly, the limited number of CNVs mapped in the BrCI and ErRo had as a consequence an effect on the number of CNVRs identified in these two breeds. Interestingly, [18] found that in the Mexican turkey population, a backyard population not under selection, the number of CNV and CNVRs was much higher respect to the population under selection. These results suggest that the CNVs variability (size, number, and state) may be related to the different breeding strategies and selection goals underwent in these populations. Most likely, these two populations have been strongly selected in inbreeding mating to fix morphological characteristics and have been recovered as a breed from a small number of birds.

Focusing on CNVRs state, we found that 1% of the CNVRs resulted from complex regions in a few individuals per breed, while the highest observable proportion was for gain regions (60%). The directional selection for specific traits, together to a dietary shift because of domestic farming practices with the subsidy of feed, may have affected the increase in copies for specific genes, as occurred in dogs [37] or polar bears [38], where a dietary shift produced an increase in DNA copy number of the AMY gene involved in metabolism of starch. In livestock populations, where there is no strong directional selection, the proportion of complex CNVRs is higher, as found Mexican Creole chickens (14% of complex CNVRs) and in Mexican Creole cattle (16% of complex regions) [36,39], where this proportion was up to 16 and 14%, respectively. Contrariwise, in the avian species where a strong selection occurred as in pure lines for the production of hybrids or in turkey purebreds, a very low proportion of complex CNVRs, 0% to 5%, was found [18,40].

Excluding singleton_CNVRs, only 38 regions of the remaining CNVRs (n. 119) harbored protein-coding genes (total number of annotated gene = 54). The available annotation for protein-coding gene in turkey is still at its infancy with respect to other annotation as in chicken or other species. Furthermore, the genetic basis of specific phenotypes such as brooding aptitude (mainly in BrCI considered the best breed for this reproductive characteristic), adaptation to the harsh environment and hardiness, characterizing all the breeds here analyzed, are poorly studied in turkey and also in the most similar species of chicken. In fact, for most of the annotated genes, we did not find any specific association with function or traits directly studied in turkey populations, but, as reported in Tables S2 and S3, most of the traits related to our genes have been previously studied in other species such as chicken, pig, bovine, birds, mice, zebrafish, and human. In addition, the Animal Genome Chicken Database did not reveal any relationships between genes annotated in the CNVRs here identified and QTL except for the *ESR1* gene, which was found associated with the eggshell thickness (QTL:11828).

### 4.1. Breed_CNVRs

One hundred and one breed_CNVRs have been identified with a variable number for each turkey breeds ranging from 2 in all breeds to 19 in RoMa. When we consider breed_CNVRs identified in at least 10 individuals, this number decreases to one for all breeds except for RoMa, which was three. These regions were identified with the same CNVR state in all samples. In the BrCI’s CNVR_051, identified in 11 birds as a loss, the *ESR1* gene involved in the maintenance and function of the shell gland [41] was annotated, while *CHID1* involved in beef marbling [42] was included in the region mapped in ErRo (CNVR_099). Additionally, within the CNVR_027 (a loss region for 13 PrPc birds), two genes were annotated: the *GUCY1A2* gene involved in immunity, having a role in regulating the proliferation and elimination of T cells and maintaining its number stable in the absence of external stimulus [43], and the *VMO1* gene, one of the protein components of the outer layer of vitelline membrane, which plays an essential role as antimicrobial barrier in avian eggs [44]. Finally, the drip loss and the power athlete status are the traits associated with the genes (*TIMM21* [45] and *CNDP2* and *CNDP1* [46], respectively) and lay within the duplicated region identified in 15 individuals of the RoMa breed (CNVR_071).

### 4.2. Shared_CNVRs

Among the 119 CNVRs identified here, only 18 were shared_CNVRs by at least two breeds. Three CNVRs (CNVR_092, CNVR_093, and CNVR_096) were in common among all the breeds, two of which (CNVR_092 and CNVR_093, both loss regions) were shared by a large number of birds (43% and 40%, respectively), including all 24 ErRo animals. No genes were annotated in these two regions. The CNVR_096 showed variability in terms of CNVR state: BrCI—loss, ErRo—gain, PrPc—loss, and RoMa—complex. Within this region is mapped the *CD8A* gene, which is known to have a role in the host immune and inflammatory response in chickens [47].

### 4.3. Comparison with Literature

When compared with the other three Italian breeds analyzed, among the 119 CNVRs here identified in at least two samples, 38 resulting regions mapped also in the BR, CoEu, and NI Italian breeds (Table S5). The breed_CNVRs (n. 101) identified here decreased to 75 when considering a larger number of birds in the joint analysis with the literature data, reflecting the importance of ameliorating existing mapping in small local populations. Interestingly the more represented CNVRs remain the CNVR_092, CNVR_093, and CNVR_096, shared by 108, 102, and 57 birds of all seven breeds, respectively.

When we consider the shared_CNVRs in two breeds (n. 53 in total), NI and RoMa shared the highest number of CNVRs (n. 6): CNVR_005, CNVR_007, CNVR_011, CNVR_043, CNVR_097, and CNVR_104. These regions, all loss, are shared by a variable number of samples (from 5 to 17) and harbored genes (*TBC1D15, LOC10490951,* and *GPHN*) that have not yet been associated with any function that is appreciable for traits of interest in livestock species. In addition, the CNVR_036 (all loss 22 birds of 5 breeds) mapped on chr11 harbors two genes *LIPG* and *ACAA2,* which both have a role in the regulation of fatty acids metabolism. The *LIPG* gene regulates and hydrolyzes serum high-density lipoprotein (HDL) to generate free fatty acids and low-lipid apolipoprotein (Apo) A1 [48]. The *ACAA2* gene is a key enzyme of fatty acid oxidation steps (β-oxidation) and was among the upregulated genes found in meat chicken (top DE genes) with respect to layers and cross (meat x layers) chickens [49]. In addition, the *LMAN1* gene is included within the same CNVR_036, and its possible role in feed efficiency and in daily occupation time in pigs have been reported by [50]. The CNVR_014 and CNVR_114 are also two regions shared by three (PrPc, CoEu, and NI) and two breeds (PrPc and CoEu), in which genes involved in some phenotypic characteristics were found. These genes also contribute to productive features in turkey: eggshell calcified layer (*OVSTL*) [51] and feed efficiency (*PRKG1*) [52]. Finally, the CNVR_032, mapped in BrCI as well as in CoEu and NI breeds, harbors the *HNRNPL* gene, which results in involved in spermatogenesis [51], i.e., a reproductive characteristic important for species survival.

The PCA performed using all seven Italian breeds, reveals important aspects related to the intra-breeds relationships. As shown in Figure 3b, the distribution of the analyzed breeds, once again reveals that the phenotypical selection feather color may have played a pivotal role in breeds’ differentiation, as the geographical origin of each breed. Birds with recessive feather colours, cluster very closely, grouping both for PrPc and ErRo in two subpopulations on PC_1. Considering the geographical origin, the distribution of turkeys from the Northern Italy regions under the Austro-Hungarian Empire domination (i.e., NI, ErRo, and BR) separate from birds of Central Italy (PrPc and RoMa), while the BrCI appears to be, as expected, in between the two clusters. Interestingly, the CoEu birds that are characterized by small body size group together in a well-separated cluster as shown in the 3D PCA graphical representation of Figure S1.

## 5. Conclusions

CNVs and CNVRs supply informative objective tools at genome level for traditional turkey breeds conservation: an accurate knowledge of the birds used in conservation plans is a basic step in the improvement of conservation projects and in the investigation of their genetic potential for their possible consideration in selection strategies for commercial hybrids. The genomes of the analyzed birds reveal the effects of breed-specific breeding strategies and the importance that the morphological traits had in local breeds selection and now in their conservation to maintain the standard described in the Autochthonous Italian Poultry Breeds Registry. The TuBAvI project collected individuals of small local Italian populations. Even if the size of the sampling may be smaller compared to research studies on commercial populations, this study represents an important step forward in the state of the art of local Italian turkey breeds’ genetic variability. Differences have been described at the genomic level related to physiological, reproductive, and behavioral traits. The analyzed breeds show high clustering properties at the genomic level, and their relationships are strictly linked to the geography and the historical farming systems of their native regions. Genome-based information about the uniqueness of the Italian local turkey breeds have been reported.

## Figures and Tables

**Table 1:** Description of breeds included in the study. Weight at adult age.

Breed (Code; n. of Birds)	Region of Origin	Weight Male (Kg)	Weight Female (Kg)	Feather and Skin Colour	Features
Bronzato Comune (BrCI; 24)	Northern Italy	6.0–7.0	3.0–3.5	Bronzed. White Skin.	Rustic breed; strong hatching attitude; breeding in local areas
Ermellinato di Rovigo (ErRo; 24)	Veneto	10.0	4.0–5.0	Black-laced/White. White Skin.	Rustic breed; slow growing excellent grazers; breeding in local areas
Parma e Piacenza (PrPc; 25)	Emilia-Romagna	12.0	6.5	Steel Gray/Dull Black. White Skin.	Local breeding; numerical consistency extremely small.
Romagnolo (RoMa; 30)	Emilia-Romagna	5.0–6.0	3.0–4.0	Different colors. Yellow skin.	Rustic breed; excellent grazers; breeding in local areas.

**Table 2:** Descriptive statistics of CNVs identified in each turkey Breed. CNV length are expressed in base pairs (bp).

Breed	n. CNVs	n. CNV per Sample Min - Max (Mean)	Loss	Gain	Loss/Gain	Min Length	Max Length	Mean Length
BrCI	223	6–19 (9)	162	61	2.65	819	185,488	9123
ErRo	177	4–13 (7)	130	47	2.76	1227	195,656	9148
PrPc	341	6–42 (14)	151	190	0.79	1278	230,199	18,791
RoMa	336	5–24 (12)	191	145	1.32	688	356,195	17,748

**Table 3:** Summary of CNVRs mapped in each turkey breed. All lengths are expressed in base pairs (bp).

Breed	n. CNVRs	Loss	Gain	Complex	Singleton ^1^	Min Length	Max Length	Mean Length	Total Coverage (%) ^2^
BrCI	89	47	42	0	51 (57.3)	819	185,488	11,612	1,033,536 (0.11)
ErRo	50	25	25	0	30 (60)	1227	195,656	13,597	679,899 (0.07)
PrPc	225	67	156	2	184 (72.2)	1278	230,199	12,969	2,918,067 (0.32)
RoMa	155	62	91	3	106 (68.4)	688	451,214	13,413	2,079,015 (0.23)

^1^ (% on total CNVR number); ^2^ Proportion calculated on total *Meleagris gallopavo* (GCA_000146605.4; Turkey_5.1) autosomes length (903.48 Mb).

**Table 4:** List of CNVRs mapped in at least 10 animals. For each CNVR, the number of birds per breed is reported together with their total and the annotated genes.

CNVR_ID	Chr	Start	End	CNVR State	BrCI	ErRo	PrPc	RoMa	Tot Birds	Genes
CNVR_017	1	98,886,764	98,931,838	loss	9	3	5		17	
CNVR_027	1	175,858,843	176,089,042	loss			13		13	*VMO1, GUCY1A2*
CNVR_045	2	14,079,733	14,082,532	loss			2	9	11	
CNVR_050	2	42,604,981	42,606,860	loss	12			6	18	
CNVR_051	2	47,933,799	47,937,842	loss	11				11	*ESR1*
CNVR_058	2	101,084,671	101,087,053	loss	16	7			23	
CNVR_069	3	20,396,386	20,399,251	loss	13		22	3	38	
CNVR_071	3	37,360,746	37,811,960	gain				15	15	*LOC104910234, C3H18orf63, LOC100540739, TIMM21, DIPK1C, CNDP2, CNDP1, ZNF407*
CNVR_092	4	63,830,569	63,837,531	loss	5	24	2	13	44	
CNVR_093	4	63,850,913	63,854,111	loss	6	24	2	9	41	
CNVR_096	4	68,473,939	68,512,066	complex	6	13	3	6	28	*CD8A*
CNVR_099	5	14,373,860	14,376,729	loss		16			16	*CHID1*
CNVR_100	5	14,780,699	14,782,967	loss	6		8	6	20	*TSPAN4*
CNVR_036	11	18,811,230	19,015,763	complex	5	3	4		12	*LOC104912696, C11H18orf32, LIPG, ACAA2, LMAN1, LOC100543122, CPLX4*
CNVR_060	21	4,564,447	4,569,014	loss				12	12	
CNVR_061	21	5,878,926	5,903,943	loss				19	19	
CNVR_064	28	2,025,784	2,027,311	loss	9	5			14	

## Data Availability

The data presented in this study are available on request from the corresponding author. The data are not publicly available due to project IP rules.

## Supplementary Materials

The following are available online at https://www.mdpi.com/2076-2615/11/2/391/s1, Table S1: List of CNVs identified in all Italian turkeys. Table S2: List of CNVRs identified in the four Italian turkeys together with the annotated genes; Table S3: List of breed_CNVRs identified in at least 2 birds (sheet1); References related to sheet1 (sheet 2). Table S4: Annotation performed using DAVID Database; Table S5: List of CNVR regions identified in seven Italian Turkey breeds. Figure S1: 3 dimensions PCA result for the seven breeds.
