# Supplementary figures and images for: Copy Number Variants in Four Italian Turkey Breeds
Source: Animals (Basel). 2021 Feb 3;11(2):391. doi: 10.3390/ani11020391 (PMC7913726; doi:10.3390/ani11020391)

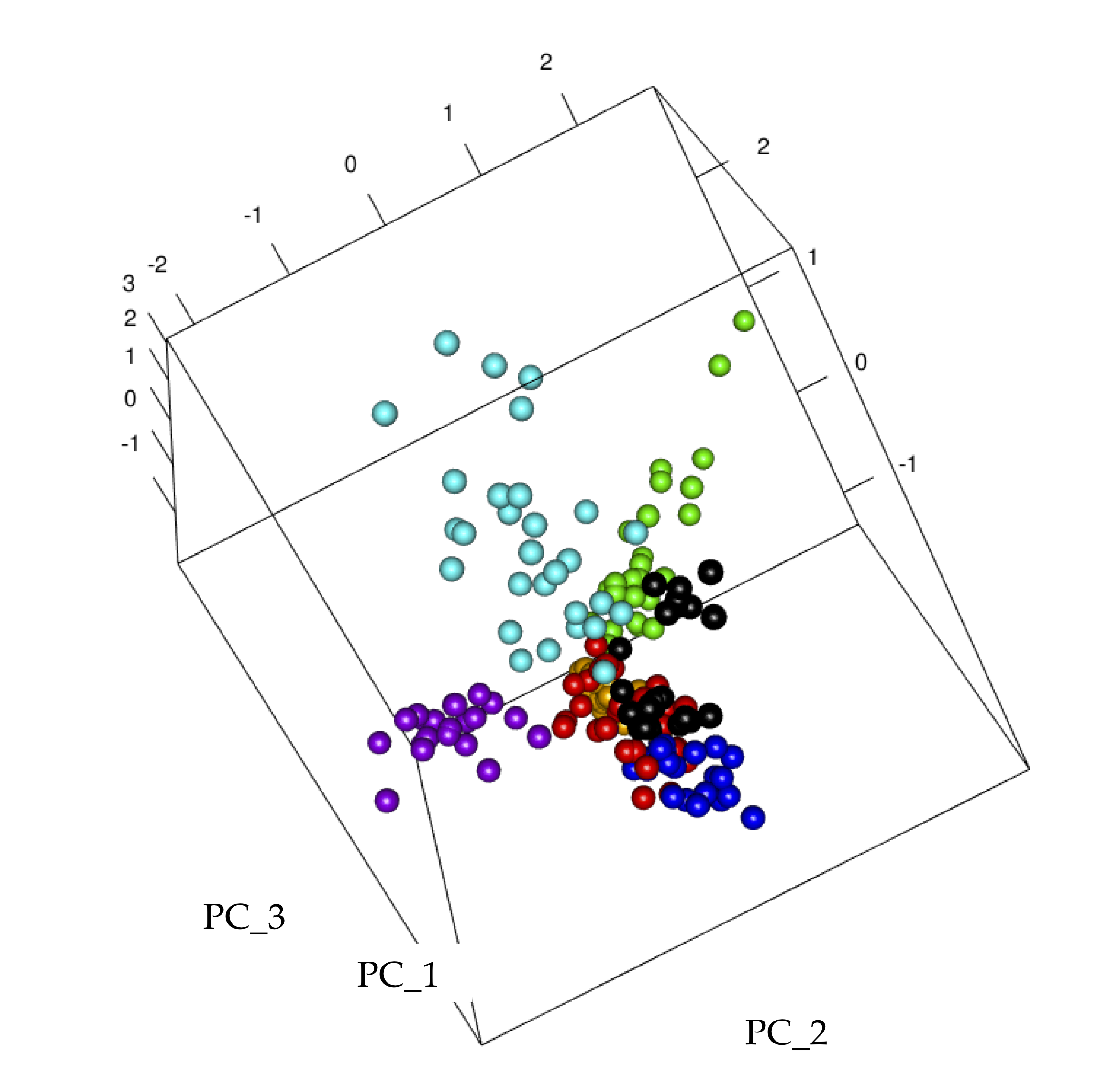

Supplement: Supplementary file 1 [file animals-11-00391-s001.zip › Supplementary_Tables_Figures/Figure_S1.tiff]
